# Early Diagnosis and Treatment in Infective Endocarditis: Challenges for a Better Prognosis

**DOI:** 10.5935/abc.20180270

**Published:** 2019-02

**Authors:** Daniely Iadocico Sobreiro, Roney Orismar Sampaio, Rinaldo Focaccia Siciliano, Calila Vieira Andrade Brazil, Carlos Eduardo de Barros Branco, Antônio Sergio de Santis Andrade Lopes, Flávio Tarasoutchi, Tânia Mara Varejão Strabelli

**Affiliations:** 1 Unidade Clínica de Cardiopatias Valvares do Instituto do Coração do Hospital das Clínicas da Faculdade de Medicina da Universidade de São Paulo, São Paulo, SP - Brazil; 2 Unidade de Controle de Infecção Hospitalar do Instituto do Coração do Hospital das Clínicas da Faculdade de Medicina da Universidade de São Paulo, São Paulo, SP - Brazil

**Keywords:** Endocarditis, Bacterial/mortality, Prosthesis Implantation, Catheters, Pacemaker, Artificial, Diagnostic Imaging, Echocardiography

Infective endocarditis (IE), a microbial infection of the cardiac or adjacent vascular endothelium, remains a feared disease, although the modern diagnosis systematizations date back to 1885 by Osler.^1^ Although relatively uncommon,^2^ with approximately 3-10 cases per 100,000 individuals/year,^3^ the mortality remains high: more than one-third of patients die in the first year after the diagnosis.^1,4^ Only early diagnosis and treatment, whether exclusively clinical or associated with cardiac surgery, may interfere to reduce this high mortality rate.

IE used to be more frequent in young and middle-aged adults with rheumatic or congenital heart disease.^3^ However, recent studies have shown a significant reduction in the incidence of IE in these groups, especially in more developed countries.^2^

IE can be increasingly seen in patients with valve prostheses, vascular catheters, implantable electronic devices such as pacemakers and implantable cardiac defibrillators^5,6^ and new surgical devices, such as transcatheter valve implantation.^2^ Moreover, due to the population aging, even in Brazil, an increased incidence has been observed in the elderly, especially when associated with comorbidities such as diabetes (20%), chronic kidney disease (14%) and anemia (10%),^5^ with a 4.6-fold increase in IE, when compared to the general population.^5,6^ At the same time, reflecting the change in the epidemiology, the incidence of endocardial infection by staphylococci has been steadily increasing, even predominating in relation to streptococci in many centers.^3,7^

The diagnosis of IE is based on the modified Duke Criteria for Infective Endocarditis: the association of clinical signs (such as fever and presence of murmur in patients with risk of heart disease), positive blood culture for frequent etiological agents and typical echocardiographic findings (vegetation, periannular abscess)^4^ show high sensitivity (> 80%), mainly in native valve infections.^4,6^ However, the criteria show lower diagnostic accuracy for an early diagnosis in clinical practice, mainly in the previously mentioned group of patients, in which the incidence has been increasing. The diagnosis is challenging, especially if the echocardiography is normal or inconclusive, as it occurs in up to 30% of cases,^8^ or when blood cultures are negative.^4,6^

In fact, negative blood cultures occur in approximately 2% to 20% of cases of endocarditis. Common causes are: concomitant or prior use of antibiotics and presence of slow-growing or difficult-to-detect microorganisms in routine cultures. The following microorganisms stand out: *Coxiella burnetii*, *Bartonella* species and fungi.^4^

**Figure 1:**
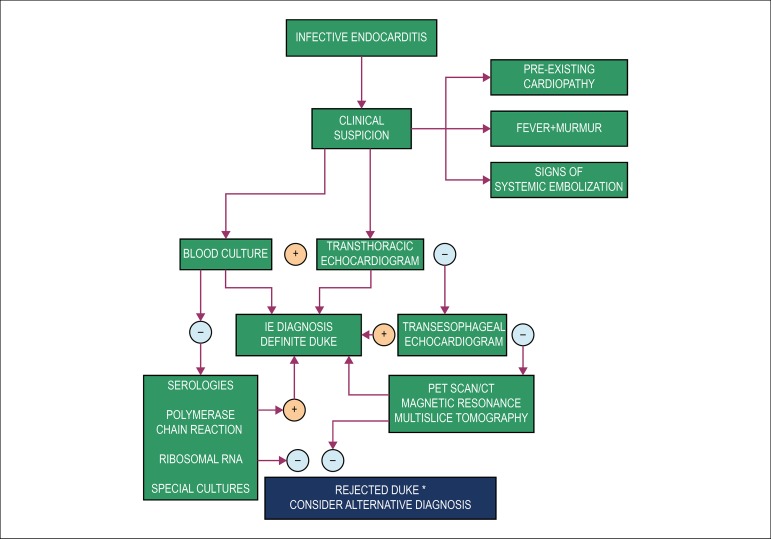
IE diagnosis flowchart. *Possible cases according to Duke’s criteria are all those that do not fit as definite or rejected cases.

The incidence of negative blood cultures has been reduced^3^ with automated blood culture techniques, specific serologies (*Coxiella sp*) and polymerase chain reaction (PCR). These methods^2^ allow the direct identification of bacterial species, especially in difficult-to-recognize cases, helping to attain an early diagnosis in relation to routine culture methods.^3^ (Figure 1)

Imaging methods, mainly echocardiography, play a key role in the diagnosis and management of IE.^6^ Being the technique of choice for the initial investigation, it should be rapidly performed, and if the clinical suspicion persists in the transthoracic modality, the transesophageal assessment should be carried out, with an evident increase in the method accuracy.

Individuals with prostheses and catheters or devices often require assessment by transesophageal echocardiography (TEE), considering that the sensitivity and specificity rates are between 40-70% for transthoracic echocardiography (TTE) and 85% for TEE in prosthetic valves.^8^ A negative result in the TEE does not exclude IE in patients with strong clinical suspicion. Therefore, the examination should be repeated within seven days for diagnostic clarification, whenever there is the possibility of IE.

The echocardiographic diagnosis may be limited by acoustic shadowing, confusing images, especially in the postoperative period, very small vegetation or absence of vegetation.^1^ These limitations led to a growing interest in the use of other imaging modalities that would complement the echocardiography.^9,10^

The use of transesophageal three-dimensional echocardiography has improved the evaluation of cardiac volumes and structures, mainly for better identification of paraprosthetic regurgitation. This technique has improved and will certainly be even more useful in the near future.^8^

Other imaging methods have also shown to be promising in the early diagnosis of patients with suspected IE that is difficult to be confirmed, such as multislice computed tomography (MSCT), magnetic resonance imaging (MRI) and positron-emission computed tomography (PET/CT).^1^

PET/CT has been shown to be particularly important in cases of patients with valve prostheses or cardiac devices with more than three months of implantation (Figure 1), in addition to the relevant potential in detecting extracardiac infectious foci, malignancy, and other types of inflammation.^7,9^

When assessing prosthetic valve dysfunction, a recent study^6^ suggested that MSCT may be equivalent or superior to the echocardiography to identify prosthesis-related vegetation, abscesses, pseudoaneurysms and dehiscence. However, there have been few studies comparing the two techniques and, therefore, the echocardiogram persists as the first-choice method in the investigation.^6^ Thus, it is worth emphasizing that even the most modern imaging techniques are not always conclusive or unquestionably clarify the presence of endocarditis, particularly in these difficult-to-diagnose subgroups, such as the elderly and patients with implantable devices/catheters.

In conclusion, the trinomial high clinical suspicion, microbiological and imaging methods remain essential for the early diagnosis in IE. The inclusion of new imaging and microbiological identification methods, associated to a multidisciplinary team consisting of cardiologists, infectologists, imaging specialists, microbiologists and other specialties, for specific cases, such as neurologists are crucial in this scenario.^6^

We emphasize that the change in the course of IE prognosis depends on the rapid establishment of targeted therapy, which in turn is only possible when an early diagnosis is attained.^3^ High-risk subgroups, such as the elderly and patients with implanted prosthetic material deserve special attention, as a delayed diagnosis has led to increased mortality. Thus, future guidelines should consider the inclusion of these new techniques in the diagnosis of IE.^2^
